# Validating Simple Modifications to the Rome IV Criteria for the Diagnosis of Irritable Bowel Syndrome in Secondary Care

**DOI:** 10.1111/apt.18363

**Published:** 2024-10-28

**Authors:** Vivek C. Goodoory, Christy Riggott, Mais Khasawneh, Christopher J. Black, Alexander C. Ford

**Affiliations:** ^1^ Leeds Gastroenterology Institute St. James's University Hospital Leeds UK; ^2^ Leeds Institute for Medical Research at St. James's University of Leeds Leeds UK

**Keywords:** accuracy, irritable bowel syndrome, Rome IV criteria, sensitivity, specificity

## Abstract

**Background:**

The Rome IV criteria for irritable bowel syndrome (IBS) may be too restrictive for clinical practice and research.

**Aims:**

To validate the Rome IV criteria and study the diagnostic performance of simple modifications to them.

**Methods:**

We collected symptom data from consecutive adults with suspected IBS seen in a single clinic. We used a reference standard to confirm IBS (presence of lower abdominal pain associated with altered stool form or frequency; no evidence of organic gastrointestinal disease after limited investigation). We applied Rome IV criteria, but also two modifications. First, we re‐incorporated abdominal discomfort but kept symptom frequency required for both abdominal pain and discomfort to at least 1 day per week. Second, we included only abdominal pain but relaxed symptom frequency back to 3 days per month. We calculated sensitivity, specificity and positive and negative likelihood ratios (LRs), with 95% confidence intervals (CIs), for each diagnostic criterion.

**Results:**

We recruited 170 patients (76.5% female, mean age 37.9 years). Sensitivity and specificity of the Rome IV criteria were 82.1% and 85.1%, respectively; positive and negative LRs were 5.51 (95% CI 2.95–11.3) and 0.21 (95% CI 0.14–0.31), respectively. Modifying the criteria by relaxing the frequency of abdominal pain to 3 days per month led to the best performance [sensitivity 90.2%, specificity 85.1%, positive LR 6.06 (95% CI 3.25–12.2), and negative LR 0.11 (95% CI 0.07–0.19)].

**Conclusions:**

The Rome IV criteria performed well in diagnosing IBS. A simple modification relaxing the required frequency of abdominal pain improved their performance.

## Graphical abstract

The Rome IV criteria performed well in this study. However, a modification to them, relaxing abdominal pain frequency back to 3 days per month, further improved performance due to improved sensitivity, but not at the expense of specificity. This could be a useful modification for future iterations to consider.

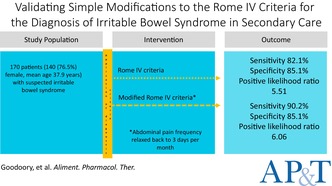

**Abbreviations**
BAD: bile acid diarrhoea
CI: confidence interval
CTC: computed tomography colonography
HADS: hospital anxiety and depression scale
IBD: inflammatory bowel disease
IBS: irritable bowel syndrome
IBS‐SSS: IBS severity scoring system
LR: likelihood ratio
PHQ‐12: patient health questionnaire‐12
SD: standard deviation
SeHCAT: 23‐seleno‐25‐homo‐tauro‐cholic acid

## 1 Introduction

Irritable bowel syndrome (IBS), characterised by abdominal pain in association with abnormal stool form or frequency [1], is a disorder of gut‐brain interaction [2], affecting 4%–10% of the general population globally [3, 4]. There is no consistent structural abnormality or reliable biomarker to support diagnosis of the condition. Recommendations are, therefore, that a positive diagnosis should be made on clinical grounds, with recourse to limited investigation [5], to exclude common organic gastrointestinal disorders, such as coeliac disease [6, 7], inflammatory bowel disease (IBD) [8], or bile acid diarrhoea (BAD) [9], which IBS may mimic. Symptom‐based diagnostic criteria were first proposed to facilitate a positive diagnosis of IBS by Manning and colleagues in the 1970s [10].

The current gold‐standard symptom‐based criteria for IBS are the Rome IV criteria, which were described in 2016 [1]. In moving from the Rome III criteria to the Rome IV criteria, the term “abdominal discomfort” was removed and the symptom frequency for abdominal pain was increased to a minimum of 1 day per week, from 3 days per month [1]. Although these changes increased the specificity of the Rome IV criteria for a diagnosis of IBS, compared with Rome III, this has come at the expense of sensitivity, with a proportion of patients felt by a clinician to have IBS no longer meeting current diagnostic criteria [11].

Validation studies of previous iterations of the Rome criteria demonstrate that they perform only modestly in diagnosing IBS [12, 13, 14, 15], although the Rome IV criteria are somewhat better in this regard [16]. Implementing recommended symptom‐based criteria is necessary to avoid over‐investigation, which patients with IBS may find anxiety‐provoking [17], but their performance should be validated independently to ensure their accuracy if they are to reassure patients and physicians that the diagnosis is secure and reduce costs of managing IBS.

We have performed an independent validation study of the Rome IV criteria previously [16], comparing their performance with the Rome III criteria for the diagnosis of IBS in over 500 patients referred with suspected IBS to secondary care. Although this confirmed that the Rome IV criteria were more specific than Rome III, they were also more accurate in terms of facilitating a diagnosis of IBS. In a longitudinal follow‐up study of these patients over 4 years, the miss rate for future organic gastrointestinal disease was only 1%, suggesting a diagnosis of IBS made using the Rome IV criteria is safe and durable [18].

It is now accepted that, despite their superior performance, the Rome IV criteria are probably too restrictive for both clinical practice and future research [19, 20]. Revisions to the Rome criteria for IBS are anticipated, although the exact changes to be made are, at present, unknown. We have studied the diagnostic performance of two simple modifications to the Rome IV criteria for IBS in the aforementioned cohort of patients [21]. First, we re‐incorporated abdominal discomfort but kept the symptom frequency required for both abdominal pain and abdominal discomfort to at least 1 day per week. Second, we included only abdominal pain but relaxed the required symptom frequency back to 3 days per month. In both modifications specificity was lower than with the Rome IV criteria but higher than Rome III. However, specificity was closer to Rome IV when only abdominal pain frequency was relaxed, but this did not come at the expense of sensitivity, which increased. This suggests that this may be a useful change to incorporate in future iterations of the Rome criteria.

It is important to point out that these modifications were assessed retrospectively and, to our knowledge, in the intervening 4 years since our study there have been no other validation studies of the Rome IV criteria. We, therefore, conducted a second independent validation study of the Rome IV criteria in a separate cohort of patients, comparing their performance with Rome III, but also assessing these two simple modifications to the Rome IV criteria, prospectively. We hypothesised that the Rome IV criteria would be superior to Rome III for the diagnosis of IBS, but that relaxing symptom frequency required for abdominal pain to only 3 days per month would further improve their performance.

## 2 Methods

### 2.1 Participants and Setting

We recruited unselected, consecutive individuals newly referred from primary care to the specialist IBS clinic in Leeds Teaching Hospitals NHS Trust, Leeds, UK with suspected IBS between September 2021 and June 2024. The hospital serves a local population of 800,000. We do not accept tertiary referrals with suspected IBS from other centres. The clinic provides a pathway to rapid diagnosis and treatment for patients suspected to have IBS referred by local primary care physicians. Two experienced consultant gastroenterologists provide their services to this clinic. There were no exclusion criteria, other than an inability to understand written English. All patients were provided with a detailed questionnaire as part of their clinical assessment at their first appointment. We obtained ethical approval to conduct this study from the Hampshire Research Ethics Committee (reference: 21/EC/0147).

### 2.2 Data Collection and Synthesis

#### 2.2.1 Demographic, Symptom and Mood Data

We collected demographic, symptom and mood data prospectively at the first clinic visit, prior to requesting any investigations that were deemed necessary. We recorded age and sex, and captured symptom data using both the Rome IV and III questionnaires for IBS in all patients [1, 22]. The presence or absence of Rome IV or Rome III‐defined IBS was assigned according to the scoring algorithms proposed for use with these questionnaires (see Table S1) [1, 23]. We evaluated IBS severity using the IBS severity scoring system (IBS‐SSS) [24], a validated seven‐item self‐administered questionnaire assessing presence, severity and frequency of abdominal pain, presence and severity of abdominal distension, satisfaction with bowel habit, and degree to which IBS symptoms are affecting, or interfering with, the person's life. It has a maximum score of 500 points, with < 75 indicating remission of symptoms, 75–174 mild, 175–299 moderate and ≥ 300 severe symptoms. We collected symptoms of anxiety and depression using the hospital anxiety and depression scale (HADS) [25]. The total HADS score ranges from a minimum of 0 to a maximum of 21 for either anxiety or depression. We categorised severity for each separately as normal (total HADS depression or anxiety score 0–7), borderline abnormal (8–10) or abnormal (≥ 11). We collected extra‐intestinal symptoms using the patient health questionnaire‐12 (PHQ‐12) [26], derived from the validated patient health questionnaire‐15 [27]. The total PHQ‐12 score ranges from a minimum of 0 to a maximum of 24. We entered all questionnaire data into an online database at the initial clinic visit, prior to referral for investigations.

#### 2.2.2 Diagnostic Work‐Up

Patients underwent relatively standardised work‐up, but investigations were minimised, wherever possible, as IBS is not a diagnosis of exclusion. We ensured all patients had a full blood count and C‐reactive protein, either at the point of referral by their general practitioner, or at their first visit to the clinic. We checked coeliac serology in all patients, irrespective of predominant bowel habit. We also checked faecal calprotectin in patients aged < 40 years with diarrhoea, with subsequent colonoscopy if ≥ 100 mcg/g. We requested colonoscopy with right‐ and left‐sided colonic biopsies in patients aged ≥ 40 years with either diarrhoea or a recent change in bowel habit. Colonoscopy, computed tomography colonography (CTC) or flexible sigmoidoscopy could be requested at the physician's discretion in other patients with atypical features, including anaemia, nocturnal diarrhoea, anorectal bleeding or weight loss. Irrespective of age, most patients with suspected IBS with diarrhoea underwent 23‐seleno‐25‐homo‐tauro‐cholic acid (SeHCAT) scanning to exclude BAD. We only classified patients with a SeHCAT retention of < 10% at 7 days as having BAD, as the response to bile acid sequestrants is best in those with moderate to severe BAD [28]. In patients with constipation with symptoms suggestive of obstructive defaecation, anorectal physiology studies were requested. Faecal elastase was requested at the discretion of the consulting doctor. Small bowel investigations, including magnetic resonance enterography or wireless capsule endoscopy, were only performed in patients with non‐specific inflammation on terminal ileal or colonic biopsies.

The clinicians performing colonoscopic examinations, radiological or physiologic investigations, or histological interpretation of biopsy specimens were blinded to questionnaire data. We defined the following as being consistent with organic disease after investigation: coeliac disease, Crohn's disease, ulcerative colitis, IBD‐unclassified, microscopic colitis, ischaemic colitis, radiation enteritis, colorectal carcinoma, BAD or exocrine pancreatic insufficiency (defined as faecal elastase < 200 mcg/g). We did not consider uncomplicated diverticular disease, colorectal adenoma, haemorrhoids or anal fissures as an organic explanation for lower gastrointestinal symptoms. We used these data to classify patients according to the presence or absence of organic gastrointestinal disease after investigation.

### 2.3 Reference Standard

We used a reference standard to define the presence of IBS for the Rome IV criteria as the presence of lower abdominal pain in association with either altered stool form or stool frequency at the first outpatient clinic appointment, in a patient who exhibited no evidence of organic gastrointestinal disease after the investigative algorithm described above. For the Rome III criteria, we used a similar definition, but also incorporated presence of abdominal discomfort. Although, to some degree, such a reference standard is an artificial construct, other studies of this type have used a similar approach [12, 14, 15, 16].

### 2.4 Statistical Analysis

We measured agreement between the Rome IV and III criteria for the diagnosis of IBS, and the reference standard, using the modified Kappa statistic, where a value < 0.2 indicates poor agreement and a value > 0.8 indicates very good agreement beyond chance. We performed these analyses using SPSS for Windows version 28.0 (SPSS Inc., Chicago, IL, USA). Our primary aim was to describe the performance of the Rome IV criteria for IBS overall, and according to IBS subtype, in evaluating the presence of IBS versus the reference standard. However, we also wanted to compare the performance of the Rome IV criteria for IBS with the previous iteration, the Rome III criteria, and assess the impact of making simple modifications to the Rome IV criteria, as we have done previously [21]. In the case of the latter, first, we re‐incorporated abdominal discomfort, if present on at least 1 day per week, and second we included only abdominal pain, but relaxed the required frequency back to 3 days per month. We calculated the sensitivity, specificity and positive and negative predictive values for each of these. We also calculated the positive likelihood ratio (LR) and negative LR, and their 95% confidence intervals (CIs). The positive LR is derived from the formula: positive LR = sensitivity/(1‐specificity), while the negative LR is derived from the formula: negative LR = (1‐sensitivity)/specificity. We performed all these analyses using StatsDirect version 3.3.6 (StatsDirect Ltd., Sale, Cheshire, England).

## 3 Results

We recruited 170 patients attending the clinic face‐to‐face during the study period, of whom 140 (76.5%) were female (age range 18–74 years; mean age 37.9 years). In total, 108 (63.5%) met the Rome IV criteria for IBS, 128 (75.3%) met the modified Rome IV criteria re‐incorporating abdominal discomfort on at least 1 day per week, 118 (69.4%) met the modified Rome IV criteria including only abdominal pain, but at a frequency of 3 days per month, and 132 (77.6%) the Rome III criteria. Characteristics of individuals meeting the Rome IV and Rome III criteria are provided in Table 1. The majority had moderate to severe symptoms, according to the IBS‐SSS, and there were high levels of mood disorders and extra‐intestinal symptom reporting, in keeping with a referral population of patients with IBS. The level of agreement, as measured using the kappa statistic, between the Rome IV and III criteria, as well as the reference standard are provided in Table 2. Agreement between the Rome IV and the Rome III criteria was good (kappa = 0.67); agreement between the Rome IV criteria and the reference standard was lower than that for Rome III. The best agreement with the reference standard was seen for the modification to the Rome IV criteria that relaxed the required frequency for abdominal pain to 3 days per month (kappa = 0.73).

**TABLE 1:** Characteristics of patients meeting the Rome IV or Rome III criteria for irritable bowel syndrome.

	Met Rome IV criteria for IBS (*n* = 108)	Met Rome III criteria for IBS (*n* = 132)
Female (%)	79 (73.1)	98 (74.2)
Mean age (SD)	36.2 (12.0)	37.7 (13.1)
Married or co‐habiting (%)	47 (43.5)	61 (46.2)
White ethnicity (%)	105 (97.2)	129 (97.7)
Smoker (%)	18 (16.7)	19 (14.4)
Alcohol use (%)	61 (56.5)	79 (59.8)
Opiate use (%)	22 (20.4)	23 (17.4)
University/postgraduate level of education (%)	40 (37.1)	54 (41.0)
Post‐infection IBS (%)	10 (9.3)	11 (8.3)
IBS subtype (%)		
IBS with constipation	21 (19.4)	12 (9.1)
IBS with diarrhoea	42 (38.9)	30 (22.7)
IBS with mixed bowel habits	44 (40.7)	90 (68.2)
IBS unclassified	1 (0.9)	0 (0.0)
Mean IBS‐SSS score (SD)	276.0 (76.8)	261.6 (83.4)
IBS‐SSS severity (%)		
Remission	1 (0.9)	3 (2.3)
Mild	10 (9.3)	19 (14.4)
Moderate	55 (50.9)	64 (48.5)
Severe	42 (38.9)	46 (34.8)
Mean HADS anxiety score (SD)	10.7 (4.9)	10.2 (4.9)
HADS anxiety (%)		
Normal	30 (27.8)	42 (31.8)
Borderline abnormal	24 (22.2)	30 (22.7)
Abnormal	54 (50.0)	60 (45.5)
Mean HADS depression score (SD)	6.8 (4.2)	6.5 (4.3)
HADS depression (%)		
Normal	64 (59.3)	81 (61.4)
Borderline abnormal	22 (20.4)	25 (18.9)
Abnormal	22 (20.4)	26 (19.7)
Mean PHQ‐12 score (SD)	10.5 (4.4)	9.8 (4.5)
PHQ‐12 severity high (%)	32 (29.6)	33 (25.0)

**TABLE 2:** Kappa statistic for levels of agreement between the Rome IV criteria, modifications to the Rome IV criteria, and the Rome III criteria and the reference standard diagnosis of irritable bowel syndrome after investigation.

	Rome IV criteria	Modified Rome IV criteria re‐incorporating abdominal discomfort on at least 1 day per week	Modified Rome IV criteria including only abdominal pain, but at a frequency of 3 days per month	Rome III criteria	Reference standard diagnosis of IBS
Rome IV criteria		0.73	0.87	0.67	0.61
Modified Rome IV criteria re‐incorporating abdominal discomfort on at least 1 day per week	0.73		0.85	0.94	0.62
Modified Rome IV criteria including only abdominal pain, but at a frequency of 3 days per month	0.87	0.85		0.79	0.73
Rome III criteria	0.67	0.94	0.79		0.70
Reference standard diagnosis of IBS	0.61	0.62	0.73	0.70	

The proportion of patients undergoing each of the diagnostic tests, and the diagnostic yield is reported in Table 3. The prevalence of organic findings after investigation in those who met the Rome IV or Rome III criteria for IBS are detailed in Table 4. No cases of coeliac disease were diagnosed in the 17 (10.0%) patients who attended without prior negative coeliac serology and required testing within the clinic. In total, 36 (21.2%) patients underwent colonoscopy and one a CTC, and 13 (7.6%) patients had a flexible sigmoidoscopy, but there were no cases of inflammatory bowel disease, microscopic colitis, or colorectal cancer detected. SeHCAT scanning was requested in 34 (20.0%) patients with suspected IBS with diarrhoea, with a retention < 10.0% in eight (23.5%) patients, seven of whom met the Rome IV criteria, six with moderate, and one severe, BAD. BAD was, therefore, the only organic diagnosis detected among patients meeting Rome IV criteria for IBS. There were a further eight (23.5%) patients with a SeHCAT retention between 10.0% and 14.9%, suggesting mild BAD, five of whom also met Rome IV criteria for IBS. Although four patients meeting criteria for IBS with constipation were diagnosed with defaecatory disorders following anorectal physiology studies, all were felt to coexist with a diagnosis of IBS. The initial diagnosis of IBS was, therefore, not revised in any of these patients.

**TABLE 3:** Investigations requested in patients with suspected IBS.

Investigation	Total number of patients (*n* = 170)	Number with organic disease
Anorectal physiology studies	6 (3.5)	4 (66.7)^a^
Coeliac serology (%)	17 (10.0)	0 (0.0)
Colonoscopy or CTC (%)	37 (21.8)	0 (0.0)
Flexible sigmoidoscopy (%)	13 (7.6)	0 (0.0)
SeHCAT scan (%)	34 (20.0)	8 (23.5)^b^
Faecal elastase (%)	2 (1.2)	0 (0.0)

^a^ All these patients were also felt to have IBS with constipation and the diagnosis was not revised in any individual.

^b^ There were a further eight patients with mild BAD (SeHCAT retention between 10.0% and 14.9%); if these individuals are included the proportion increases to 47.1%.

**TABLE 4:** Prevalence of organic disease in patients meeting the Rome IV or Rome III criteria for irritable bowel syndrome.

	Met Rome IV criteria for IBS (*n* = 108)	Met Rome III criteria for IBS (*n* = 132)
Total with organic disease (%)	7 (6.5)	7 (5.3)
IBD (%)	0 (0.0)	0 (0.0)
Microscopic colitis (%)	0 (0.0)	0 (0.0)
BAD (%)	7 (6.5)	7 (5.3)
Moderate (5.0%–9.9% retention)	6	6
Severe (< 5.0% retention)	1	1
Exocrine pancreatic insufficiency (%)	0 (0.0)	0 (0.0)

The mean age of the 108 individuals meeting Rome IV criteria was 36.2 years, and 79 (73.1%) were female. Among the 123 patients with a diagnosis of IBS according to the reference standard, 101 met the Rome IV criteria for IBS, giving a sensitivity of 82.1% (Table 5). Among 47 subjects without IBS according to the reference standard, 40 did not meet the Rome IV criteria, giving a specificity of 85.1%. The positive LR of the Rome IV criteria for the diagnosis of IBS was 5.51 (95% CI 2.95–11.3), while the negative LR was 0.21 (95% CI 0.14–0.31). When performance of the Rome IV criteria was assessed according to subtype, they performed better in predicting a diagnosis of IBS in patients with IBS with constipation (positive LR = 22.4; 95% CI 3.00–214) or IBS with mixed bowel habits (positive LR = 28.5; 95% CI 3.65–273).

**TABLE 5:** Sensitivity, specificity, positive and negative predictive values, and positive and negative likelihood ratios for the Rome IV criteria, Rome IV criteria by subtype, modifications to the Rome IV criteria, and the Rome III criteria for irritable bowel syndrome.

	Sensitivity (95% CI)	Specificity (95% CI)	Positive predictive value (95% CI)	Negative predictive value (95% CI)	Positive likelihood ratio (95% CI)	Negative likelihood ratio (95% CI)
Rome IV criteria for IBS	82.1% (74.2%–88.4%)	85.1% (71.7%–93.8%)	93.5% (87.1%–97.4%)	64.5% (51.3%–76.3%)	5.51 (2.95–11.3)	0.21 (0.14–0.31)
Rome IV criteria for IBS with constipation	86.0% (66.3%–96.5%)	96.2% (69.3%–100.0%)	97.7% (80.7%–100.0%)	78.1% (50.9%–94.4%)	22.4 (3.00–214)	0.15 (0.05–0.34)
Rome IV criteria for IBS with diarrhoea	76.1% (61.2%–87.4%)	56.3% (29.9%–80.3%)	83.3% (68.6%–93.0%)	45.0% (23.1%–68.5%)	1.74 (1.08–3.35)	0.43 (0.22–0.86)
Rome IV criteria for IBS with mixed bowel habits	84.0% (71.3%–92.6%)	97.1% (75.6%–100.0%)	98.9% (90.1%–100.0%)	66.0% (44.5%–83.6%)	28.5 (3.65–273)	0.17 (0.09–0.29)
Modified Rome IV criteria re‐incorporating abdominal discomfort on at least 1 day per week	91.9% (85.6%–96.0%)	68.1% (52.9%–80.9%)	88.3% (81.4%–93.3%)	76.2% (60.6%–88.0%)	2.88 (1.98–4.52)	0.12 (0.06–0.22)
Modified Rome IV criteria including only abdominal pain, but at a frequency of 3 days per month	90.2% (83.6%–94.9%)	85.1% (71.7%–93.8%)	94.1% (88.2%–97.6%)	76.9% (63.2%–87.5%)	6.06 (3.25–12.2)	0.11 (0.07–0.19)
Rome III criteria for IBS	92.6% (86.8%–96.4%)	80.0% (63.1%–91.6%)	94.7% (89.4%–97.8%)	73.7% (56.9%–86.6%)	4.63 (2.57–9.23)	0.09 (0.05–0.17)

Re‐incorporating abdominal discomfort on at least 1 day per week, increased sensitivity to 91.9%, but decreased specificity to 68.1%, giving a positive LR of 2.88 (95% CI 1.98–4.52) (Table 5). Including only abdominal pain, but relaxing frequency to 3 days per month, increased sensitivity to 90.2% but not at the expense of specificity, which remained 85.1%, giving a positive LR of 6.06 (95% CI 3.25–12.2).

Among the 135 patients with a diagnosis of IBS according to the reference standard, which also incorporated presence of abdominal discomfort, 125 met Rome III criteria for IBS, giving a sensitivity of 92.6% (Table 5). Among 35 subjects without IBS according to the reference standard, 28 did not meet the Rome III criteria, giving a specificity of 80.0%. The positive LR of the Rome III criteria for the diagnosis of IBS was 4.63 (95% CI 2.57–9.23), while the negative LR was 0.09 (95% CI 0.05–0.17).

## 4 Discussion

We once again validated the Rome IV criteria independently in secondary care, comparing them with the Rome III criteria but, on this occasion, we assessed the diagnostic performance of two simple modifications to them prospectively. As in our previous study, we used an accepted reference standard, of symptoms compatible with IBS, reported during the clinical history, and no organic gastrointestinal disease uncovered after a relatively standardised work‐up. As observed previously, the Rome IV criteria were more specific than Rome III, but less sensitive. However, the positive LR was higher than with Rome III, at 5.51 compared with 4.63. This means if a patient with suspected IBS meets the Rome IV criteria, they are over five times more likely to have IBS than to not have IBS. As we have observed previously, the Rome IV criteria were more accurate, in terms of the positive LR, in patients with IBS with constipation or mixed bowel habits, compared with those with IBS with diarrhoea. In fact, all cases of organic gastrointestinal disease were found in those with IBS with diarrhoea, with seven patients found to have moderate or severe BAD. We also examined the diagnostic performance of re‐incorporating abdominal discomfort, if present on at least 1 day per week, to the Rome IV criteria or including only abdominal pain, but relaxing the required frequency back to 3 days per month. The re‐incorporation of abdominal discomfort led to a worsening in performance, relative to the Rome IV criteria themselves, whereas relaxing abdominal pain frequency back to 3 days per month meant that if a patient with suspected IBS met these modified Rome IV criteria, they were over six times more likely to have IBS than to not have IBS. In a secondary or tertiary referral population in a University Hospital practice with a prevalence of IBS of 50% or more, the Rome IV criteria would identify IBS with a post‐test probability of 85% and the modification to the Rome IV criteria that relaxed abdominal pain frequency back to 3 days per month with a post‐test probability of 86%.

We recruited face‐to‐face referrals with suspected IBS, so the results of our study are likely to be generalisable to many patients in secondary care. We implemented a relatively standardised work‐up, with all patients screened for coeliac disease, either prior to attending clinic or at their first consultation, a faecal calprotectin to exclude IBD in those aged < 40 years with diarrhoea, a colonoscopy with random colonic biopsies in those with diarrhoea or a recent change in bowel habit aged ≥ 40 years, and a colonoscopy, CTC or flexible sigmoidoscopy if atypical features, such as anaemia, nocturnal diarrhoea, anorectal bleeding or weight loss, were present. In addition, most patients with suspected IBS with diarrhoea underwent SeHCAT scanning to exclude BAD. Lastly, the study followed the STARD guidelines for diagnostic accuracy studies [29], because we recruited consecutive patients, blinded assessors, and used an accepted reference standard.

Weaknesses of the study include the fact that, partly due to the study commencing shortly after the COVID‐19 pandemic, many of our clinic consultations with patients were still conducted over the telephone. This explains why the study population was smaller, at 170 patients, than our previous study [16]. Although the study was conducted in secondary care, the setting was a single specialist IBS clinic. Hence, the patient population is likely to be enriched for a diagnosis of IBS, and the Rome IV criteria and the modifications to them we describe may not perform as well in general gastroenterology clinics. We did not mandate an exhaustive diagnostic work‐up to exclude organic disease in all individuals as part of the study design because current UK guidelines for the management of IBS do not recommend this approach [5]. Faecal calprotectin could be less sensitive for the detection of small bowel Crohn's disease, although meta‐analyses suggest this is not the case [30, 31]. We only checked a faecal elastase in two patients. UK management guidelines do not recommend this is requested routinely in all patients with chronic diarrhoea [32], only if fat malabsorption is suspected. This may mean that some organic gastrointestinal disease has been overlooked, although we feel this is unlikely. We have shown previously that a diagnosis of IBS using the Rome IV criteria and limited judicious investigation is durable during longitudinal follow‐up, with only 1% of patients found to have organic gastrointestinal disease subsequently [18].

Meta‐analyses and diagnostic accuracy studies of all previous symptom‐based criteria for IBS have demonstrated only modest performance of prior criteria, such as Manning, Rome I, Rome II and Rome III [12, 14, 15, 33]. The Rome IV criteria outperformed the Rome III criteria for the diagnosis of IBS in this study and a previous study [16], due to their increased specificity, but they are probably too restrictive for both research and clinical practice [19, 20], as reflected by their lower sensitivity for the diagnosis of IBS. The Rome V criteria are in progress, although the changes that will be made in moving from Rome IV to Rome V are uncertain at the present time. Our study provides further evidence that relaxing abdominal pain frequency back to 3 days per month does not impact the overall performance of the Rome IV criteria and, if anything, improves it. This is because sensitivity increases, but specificity is more or less maintained at this frequency threshold for abdominal pain [21]. This approach is, therefore, worthy of consideration for future iterations of the Rome criteria.

This study also demonstrates that if the Rome IV criteria were to be applied in combination with routine blood tests, coeliac serology and faecal calprotectin to make a positive diagnosis of IBS in routine practice, the rate of missed organic gastrointestinal disease would be very low. The only organic disease detected in this study was BAD in seven patients with IBS with diarrhoea, accounting for only 6.5% of the entire cohort. It is unclear whether an abnormal SeHCAT retention represents true BAD in patients with IBS‐D, or whether it is an epiphenomenon. To mitigate against this to some degree we used a retention < 10% to define BAD. If these patients were re‐classified to having IBS rather than BAD, there would be no cases of organic disease in the cohort and the sensitivity of the Rome IV criteria would be 83.1% and the specificity 100%. The detection of possible BAD in those with IBS with diarrhoea explains why the Rome IV criteria performed better in patients with IBS with constipation or mixed bowel habits and suggests that, in these two groups of patients, no further investigation is needed if initial bloods are normal and the Rome IV criteria are met, even though patients with IBS with mixed bowel habits may report diarrhoea. It also underlines the importance of taking a careful clinical history to distinguish between IBS with diarrhoea and IBS with mixed bowel habits. Previous studies have demonstrated that microscopic colitis is more common in females ≥ 45 years with diarrhoea [34], but we did not detect any cases among the 36 patients undergoing colonoscopy in this cohort.

To summarise, in this validation study of the Rome IV and III criteria simultaneously, together with two simple modifications to the Rome IV criteria, in a secondary care population of patients with suspected IBS, the Rome IV criteria performed better than Rome III for diagnosis of IBS. This was mainly due to their higher specificity but came at the expense of reduced sensitivity. However, a modification to the Rome IV criteria, which relaxed abdominal pain frequency back to 3 days per month, further improved performance due to improved sensitivity, but not at the expense of specificity. Our study suggests this could be a useful modification for future iterations of the Rome criteria for IBS to consider. Finally, the results of the study underlines that making a clinical diagnosis of IBS using the Rome criteria in combination with limited judicious investigation is a safe approach to adopt in clinical practice, but that BAD should be considered as a possible alternative diagnosis in patients with IBS with diarrhoea.

## Author Contributions

**Vivek C. Goodoory:** conceptualization, data curation, writing – review and editing. **Christy Riggott:** data curation, writing – review and editing. **Mais Khasawneh:** writing – review and editing, data curation. **Christopher J. Black:** conceptualization, data curation, writing – review and editing. **Alexander C. Ford:** conceptualization, writing – original draft, writing – review and editing, formal analysis, data curation.

## Conflicts of Interest

The authors declare no conflicts of interest.

## Contributor and Guarantor Information

ACF is guarantor. He accepts full responsibility for the work and the conduct of the study, had access to the data, and controlled the decision to publish. The corresponding author attests that all listed authors meet authorship criteria and that no others meeting the criteria have been omitted.

## Supporting information


Table S1.

## Data Availability

The data that support the findings of this study are available from the corresponding author upon reasonable request.
