# Supplementary material for: Validating Simple Modifications to the Rome IV Criteria for the Diagnosis of Irritable Bowel Syndrome in Secondary Care
Source: Aliment Pharmacol Ther. 2024 Oct 28;61(2):354–62. doi: 10.1111/apt.18363 (PMC11671724; doi:10.1111/apt.18363)
Supplement: Supplementary file 1 — Table S1. [file APT-61-354-s001.docx]

**Supplementary Table 1. Rome IV and Rome III Diagnostic Criteria for IBS.**

| **Rome IV IBS Diagnostic Criteria^1^** | **Rome III IBS Diagnostic Criteria^2^** |
| --- | --- |
| 1. Recurrent abdominal pain, on average, at  least **1 day per week** in the last 3 months  and associated with two or more or the  following:  a. **Related** to defaecation  b. Associated with a change in frequency  of stool  c. Associated with a change in form of  stool | 1. Recurrent abdominal pain **or discomfort** at least **3 days per month** and associated with two or more of the following:  a. **Improvement** with defaecation  b. Onset associated with a change in frequency of stool  c. Onset associated with a change in form of  stool |
| 2. Criteria fulfilled for the last 3 months with symptom onset at least 6 months prior to diagnosis | 2. Criteria fulfilled for the last 3 months with symptom onset at least 6 months prior to diagnosis |

Note: Key differences between Rome III and Rome IV are highlighted.

**REFERENCES**

1. Mearin F, Lacy BE, Chang L, et al. Bowel disorders. *Gastroenterology.* 2016;150:1393-1407.

2. Longstreth GF, Thompson WG, Chey WD, Houghton LA, Mearin F, Spiller RC. Functional bowel disorders. *Gastroenterology.* 2006;130:1480-1491.
